# Nervous System-Systemic Crosstalk in SARS-CoV-2/COVID-19: A Unique Dyshomeostasis Syndrome

**DOI:** 10.3389/fnins.2021.727060

**Published:** 2021-08-27

**Authors:** Harnadar Anand, Victoria Ende, Gurinder Singh, Irfan Qureshi, Tim Q. Duong, Mark F. Mehler

**Affiliations:** ^1^The Saul R. Korey Department of Neurology, Albert Einstein College of Medicine, Bronx, NY, United States; ^2^Renaissance School of Medicine at Stony Brook University, Stony Brook, NY, United States; ^3^Biohaven Pharmaceuticals, New Haven, CT, United States; ^4^Department of Radiology, Albert Einstein College of Medicine and Montefiore Medical Center, Bronx, NY, United States; ^5^Department of Physiology and Biophysics, Albert Einstein College of Medicine, Bronx, NY, United States; ^6^Dominick P. Purpura Department of Neuroscience, Albert Einstein College of Medicine, Bronx, NY, United States; ^7^Department of Psychiatry and Behavioral Sciences, Albert Einstein College of Medicine, Bronx, NY, United States; ^8^Institute for Brain Disorders and Neural Regeneration, Albert Einstein College of Medicine, Bronx, NY, United States; ^9^Rose F. Kennedy Center for Intellectual and Developmental Disabilities, Albert Einstein College of Medicine, Bronx, NY, United States; ^10^Einstein Cancer Center, Albert Einstein College of Medicine, Bronx, NY, United States; ^11^Gottesman Institute for Stem Cell Biology and Regenerative Medicine, Albert Einstein College of Medicine, Bronx, NY, United States; ^12^Center for Epigenomics, Albert Einstein College of Medicine, Bronx, NY, United States

**Keywords:** autonomic nervous system, COVID-19 sequelae, evolutionary processes, premature aging, combinatorial therapeutics

## Abstract

SARS-CoV-2 infection is associated with a spectrum of acute neurological syndromes. A subset of these syndromes promotes higher in-hospital mortality than is predicted by traditional parameters defining critical care illness. This suggests that deregulation of components of the central and peripheral nervous systems compromises the interplay with systemic cellular, tissue and organ interfaces to mediate numerous atypical manifestations of COVID-19 through impairments in organismal homeostasis. This unique dyshomeostasis syndrome involves components of the ACE-2/1 lifecycles, renin-angiotensin system regulatory axes, integrated nervous system functional interactions and brain regions differentially sculpted by accelerated evolutionary processes and more primordial homeostatic functions. These biological contingencies suggest a mechanistic blueprint to define long-term neurological sequelae and systemic manifestations such as premature aging phenotypes, including organ fibrosis, tissue degeneration and cancer. Therapeutic initiatives must therefore encompass innovative combinatorial agents, including repurposing FDA-approved drugs targeting components of the autonomic nervous system and recently identified products of SARS-CoV-2-host interactions.

## Introduction

Coronavirus Disease 2019 (COVID-19) is a systemic disease that impacts multiple organ systems and is caused by the severe acute respiratory syndrome coronavirus 2 (SARS-CoV-2) virus (Zhu et al., 2020). Like some other coronaviruses, SARS-CoV-2 is a zoonotic virus that has likely jumped from animal species to humans. The potential rapid viral evolution that occurs by transitioning between poorly defined intermediate hosts may engender novel forms of pathogen-host interactions (Zhang and Holmes, 2020). Moreover, the human central nervous system has also undergone accelerated evolutionary innovations in the hominid-to-human lineage (Mattick and Mehler, 2008; Qureshi and Mehler, 2012, 2014). While these evolutionary mechanisms have facilitated rapid change in the human neocortex, other areas of the brain involved in more caudal midline homeostatic functions, which are often also preferential viral targets, represent more primordial evolutionary centers. The interplay between the potential rapid evolution of SARS-CoV-2 and regions of the human brain that have experienced rapid and differential evolution like the neocortex may be a mechanism underlying the unique profiles of damage caused by SARS-CoV-2.

It is increasingly evident that SARS-CoV-2 is not only neurotropic, but is associated with a much broader spectrum of acute and atypical neurological syndromes and manifestations than prior infections, particularly those involving β-coronaviruses (Bohmwald et al., 2018; Desforges et al., 2019; Ellul et al., 2020; Iadecola et al., 2020; Koralnik and Tyler, 2020; Paterson et al., 2020). Severe acute neurological events observed in COVID-19 have included ischemic stroke, intracranial hemorrhage, diffuse encephalopathy, encephalitis and neuromuscular disorders (Alquisiras-Burgos et al., 2020; Lee et al., 2020; MacLean et al., 2020; Conklin et al., 2021). Neurocognitive symptoms and dysfunction of various severities have also become increasingly recognized as potential consequences of SARS-CoV-2 infection (Levine et al., 2020; Graham et al., 2021; Taquet et al., 2021). The occurrence of severe neurologic dysfunction suggests that less obvious neuropathologic processes are likely present among patients who do not exhibit overt neurologic disease but may exhibit differential degrees of systemic involvement. This raises the important question of the potential mechanistic interrelationships between nervous system and systemic homeostasis in mediating the pathogenesis and progression of COVID-19.

In recent large acute retrospective incidence studies, higher in-hospital mortality has been associated with the early presence of a subset of neurological syndromes seen with COVID-19 (Eskandar et al., 2020; Chou et al., 2021). The most commonly observed central nervous system manifestations included stroke, encephalopathy, seizures and neuro-COVID-19 complex (Chou et al., 2021). These neurological complications were observed in 82% of hospitalized patients (Chou et al., 2021). Among these manifestations, altered arousal, orientation, attention, concentration (encephalopathy) and stroke conferred a significantly higher risk of mortality, independent of overall disease severity measures as assessed by a novel integrative COVID-19 severity rating scale (Altschul et al., 2020). These observations suggest that the nervous system *writ large* plays a preeminent role in the course and outcomes of SARS-CoV-2 infection.

**FIGURE 1:**
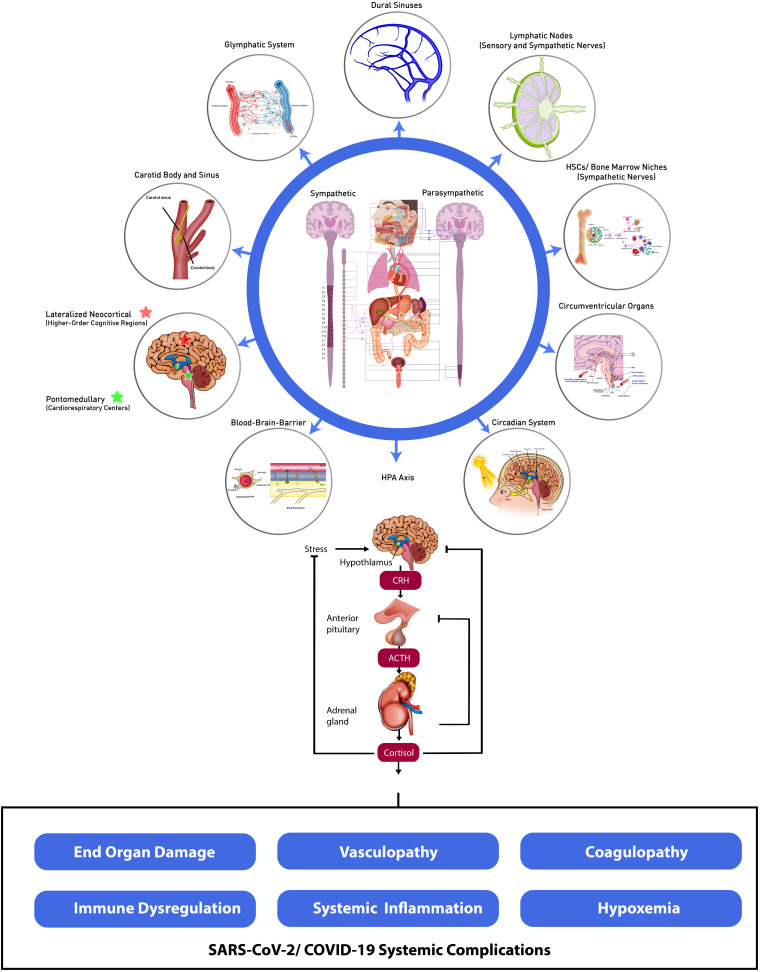
Dynamic neural-systemic interactions mediating SARS-CoV-2 infection. CSF, cerebrospinal fluid; ISF, interstitial fluid; HSCs, hematopoietic stem cells; SFO, subfornical organ; PVN, paraventricular nucleus; PBN, parabrachial nucleus; DMV, dorsal motor nucleus of the vagus; NTS, nucleus tractus solitarius; RVLM, rostral ventrolateral medulla; OVLT, organum vasculosum of the lamina terminalis; MPO, myeloperoxidase; AVP, arginine vasopressin; HPA, hypothalamic–pituitary–adrenal; CRH, corticotrophin-releasing hormone; ACTH, adrenocorticotropic hormone.

In this review, we provide emerging evidence that SARS-CoV-2 targets widely distributed components of the central and peripheral nervous systems (CNS and PNS, respectively). We propose that disruption of cardinal homeostatic mechanisms mediated by the CNS and PNS give rise to the spectrum of unanticipated, novel, and multifactorial somatic organ, tissue and cellular damage observed in COVID-19. These include often severe and frequently broad-based end-organ dysfunction, and biologically complex forms of vasculopathy, coagulopathy, hypoxemia and immune deregulation and systemic inflammation amongst other systemic and life-threatening complications (Perico et al., 2020; Nie et al., 2021). We postulate that SARS-CoV-2-induced deregulation of the central and peripheral nervous systems subvert cardinal systemic homeostatic functions through interference amongst dynamic neural and systemic organ, tissue and cellular interfaces and SARS-CoV-2-mediated cellular signaling pathways, modulatory interactions and associated multidimensional effector networks (Qureshi and Mehler, 2013; Figure 1). Characterizing such intricate nervous system-systemic crosstalk and associated viral-host evolutionary adaptations is therefore essential for identifying novel measures to address the morbidity and mortality of the acute and critical care illness as well as the long-term sequelae of SARS-CoV-2 infection.

## Evolutionary Adaptations

**FIGURE 2:**
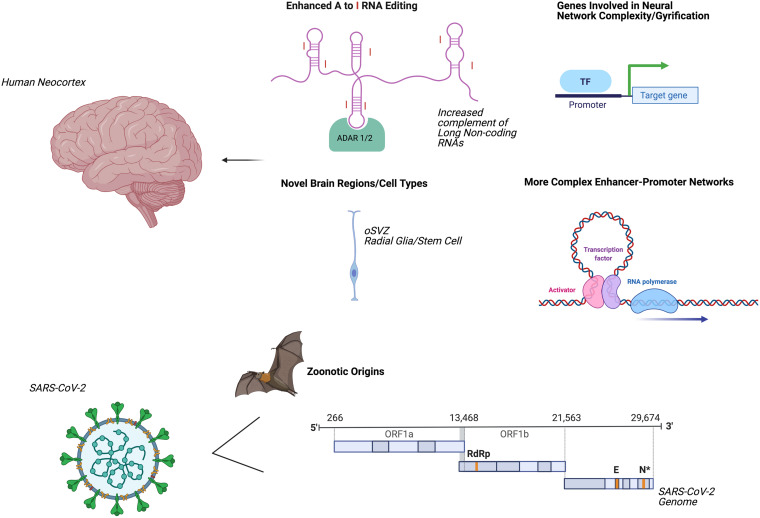
Accelerated evolution of human brain and SARS-CoV-2: potential mechanisms underlying systemic dyshomeostasis. Evolutionary adaptations mediating innovations in human brain form and function (upper panel). Parallel evolutionary adaptations in SARS-CoV-2 (lower panel). Created using biorender.com. A, Adenosine; I, inosine; ADAR, adenosine deaminases acting on RNA; oSVZ, outer subventricular zone; TF, transcription factor; ORF, open reading frame; RdRp, RNA-dependent RNA-polymerase; E, envelope protein; N, nucleocapsid protein.

Several mechanisms by which the human neocortex has undergone accelerated evolution have been elucidated (Figure 2). These innovations in regional nervous system form and function may promote higher-order cognitive and behavioral repertoires while simultaneously enhancing the vulnerability for age-related human brain disorders such as degenerative dementias and accelerated aging phenotypes (Mattick and Mehler, 2008). For example, numerous transcription factors interact with *cis*-acting genomic elements such as enhancers and super-enhancers to control the gene expression of the human forebrain (Nord et al., 2015). Degrees of adenosine to inosine RNA editing (Mehler and Mattick, 2007) of transcripts and proximal promoter associated regions with higher levels of chromatin interactivity are also enriched for human-specific genes (Song et al., 2020). Additionally, the emergence of individual human-specific genes such as *NOTCH2NL, SRGAP2, TBC1D3*, and *ARHGAP11B* may be central to increased gyrification and to the preferential activation of basal progenitors, which are thought to give rise to an expansion of the population of human neocortical neurons and to contribute to a greater sophistication of neural network connections and functional properties (Florio et al., 2015; Ju et al., 2016; Rincic et al., 2016; Fiddes et al., 2018). The expansion of the human neocortex has also been linked to evolutionary changes in neural stem cells forming the outer subventricular zone (Lui et al., 2011) and to a delay in the neuroepithelial cell differentiation process (Benito-Kwiecinski et al., 2021).

Current studies generally agree that SARS-CoV-2 likely emerged via zoonotic spillover first from bats (Lei and Zhang, 2020; Hedman et al., 2021). Other mammals have been proposed as intermediate hosts; for example, minks are susceptible to natural SARS-CoV-2 infection and can transmit the virus to humans (Hedman et al., 2021), and mutational analysis of the SARS-CoV-2 receptor-binding domains suggests that increased human susceptibility may emerge in pangolins (Lei and Zhang, 2020; Li et al., 2020a). These contingencies suggest that SARS-CoV-2 exhibits novel evolutionary trajectories, responds to selective pressures, generates genomic innovations and gives rise to innate immune dysregulation, thereby promoting greater transmissibility and more complex and interrelated clinical syndromes (Zeberg and Paabo, 2020; Zhang and Holmes, 2020). Modifications in SARS-CoV-2 cellular tropism elicit deregulated and more severe and atypical nervous system and systemic pathologies in the acute phase of illness and the contextual alterations to give rise to long-term neural and systemic sequelae (Jacob et al., 2020; Pellegrini et al., 2020). SARS-CoV-2 therefore dramatically modifies the host-mediated transcriptome and epitranscriptome, long-distance molecular signaling pathways, profiles of multi-organ cellular involvement, activation of premature aging pathways, and regenerative responses that define acute COVID-19 infection (Kim et al., 2020; Shen et al., 2020; Delorey et al., 2021). Such systemic consequences and pathogenic mechanisms give rise to a plethora of imbalanced immunological and inflammatory reactions that sculpt disease progression, severity and the trajectory of clinical inflection points (Blanco-Melo et al., 2020; Lucas et al., 2020; Meckiff et al., 2020; Su et al., 2020; Liu et al., 2021; Rodda et al., 2021). Moreover, the exceptional versatility of the lifecycle of the ACE2 receptor, SARS-CoV-2 molecular constituents, entry factors, functional adaptations and downstream signaling axes help to ensure rapid and efficient environmental innovations including linking targeted co-morbidities to cardinal viral-host mediated signaling pathways, age- and gender-dependent disease phenotypes and seminal evolutionary mechanisms, including formation of novel ACE2 isoforms through co-option of intronic retroelements as promoters and alternative exons (Anand et al., 2020; Konno et al., 2020; Maremanda et al., 2020; Shang et al., 2020; Singh et al., 2020; Wang et al., 2020b).

## SARS-CoV-2 Neurotropism

### CNS and PNS Interrelationships

SARS-CoV-2 infects the nervous system (NS) through multiple points of entry, leading to the severity and unique characteristics of systemic involvement of the virus in mediating cumulative disease burden and excessive morbidity and frequently mortality. The nervous system is composed of the CNS and the PNS. The PNS is further divided into the somatic and autonomic nervous systems. The autonomic nervous system is then segregated into the parasympathetic, sympathetic, and enteric divisions. These PNS pathways provide important communication routes to systemic organs, tissues and cell types and their molecular effectors for also defining NS viral tropism (Qureshi and Mehler, 2013; Shahriari et al., 2020; Zahalka and Frenette, 2020).

Other coronaviruses have been known to directly invade the brainstem (Porzionato et al., 2020), where the presence of the major receptor for SARS-CoV-2, angiotensin converting enzyme 2 (ACE2), has been documented. Previous studies have suggested that SARS-CoV-2 may spread to the CNS directly, by invasion through the cribriform plate into the olfactory epithelium and olfactory tract (Baig et al., 2020). The olfactory bulb has direct neuronal connections to the amygdala, entorhinal area, and hippocampus (Serrano et al., 2021). Additionally, a recent neuro-anatomical study of patients who died with COVID-19 found that 20% of patients studied had SARS-CoV-2 RNA in one or more regions of the CNS, including the olfactory bulb, amygdala, entorhinal area, temporal and frontal neocortex, and dorsal medulla (Serrano et al., 2021). In this study, the olfactory bulb was the only brain region with viral RNA in more than one subject and had the strongest PCR signal of brain regions studied (Serrano et al., 2021), suggesting that the CNS may be accessed through this route.

In addition to modulating basal homeostatic functions, the sympathetic nervous system (SNS) is responsible for “fight or flight” reactions. In such situations where the body is under stress, the SNS acts to mitigate this through global release of adrenaline and cortisol (Hanoun et al., 2015). The hypothalamus and pituitary gland, which express the ACE2 receptor, largely mediate the hormonal stress regulatory activity of the SNS via the hypothalamic-pituitary-adrenal (HPA) axis and may thus represent cardinal central viral targets (Pal, 2020). The nucleus tractus solitarius (NTS) of the brainstem, which acts as a sensory integrative center for several autonomic functions, projects onto the hypothalamus and also expresses the ACE2 receptor (Porzionato et al., 2020). Additionally, ACE2 is expressed in the carotid body, which can mediate local sympathetic activation to modulate optimal blood oxygenation and blood pressure regulation (Porzionato et al., 2020).

The enteric nervous system (ENS), a branch of the PNS, is located in the walls of the gastrointestinal (GI) tract, where it plays a key role in gut homeostasis, systemic metabolism and immunity. The parasympathetic NS closely interacts with this branch to control the basal state and digestive functions. Esposito et al. argue that SARS-CoV-2 can directly invade the ENS or the parasympathetic NS, and the virus can spread along the vagus nerve and its innervations of visceral organs, which can potentially provide another route of viral tropism (Esposito et al., 2020; Tassorelli et al., 2020). The parasympathetic pathway can then facilitate entry into the brainstem through vagus nerve synapses onto the NTS in the medulla oblongata (Li et al., 2020c). In rat studies of MERS-CoV, enteric involvement was shown to cause neurological symptoms and to precede respiratory infection (Zhou et al., 2017). Infection via GI inoculation led to higher viral loads in the CNS through feedback regulation via vagal efferents in these studies (Zhou et al., 2017). Moreover, recent work using murine models has identified a novel taxonomy involving twelve enteric neuron classes, distinguished by unique transcription factors, communication features, and functionality (Morarach et al., 2021). The enteric neuron classes develop a distinct diversification pattern through post-mitotic differentiation assisted by spatiotemporal defined landmarks (Morarach et al., 2021). These observations help distinguish the ENS from other branches of the NS and suggest potential new profiles of viral neurotropism embedded within the three-dimensional body axes. The relatively high concentration of ACE2 receptor and viral replication potential within the GI tract, along with frequently noted GI symptoms of COVID-19, emphasize the significance of ENS neurotropism (Esposito et al., 2020). In totality, the PNS will likely prove to be a key and under-appreciated feature of SARS-CoV-2 neurotropism, inclusive of all three autonomic branches and via direct and indirect target interactions.

### Cell Autonomous and Non-cell Autonomous Interplay

The ACE2 receptor is found in neurons, glial cells, astrocytes, endothelial cells, and those of the lung parenchyma alike (Doobay et al., 2007; Miller and Arnold, 2019). This route is likely cell autonomous in nature, with direct target infection via a requisite entry factor (e.g., the ACE2 receptor). While there is no consensus on which cells are directly infected, there is evidence for direct neurotropism of several of these cell types through the presence of contiguous active virions and intracellular viral RNA. For example, a human induced pluripotent stem cell-derived BrainSphere model has been employed to demonstrate SARS-CoV-2 infectivity of neuronal cells (Bullen et al., 2020). Similarly, Wang et al. have shown that human induced pluripotent stem cell-derived ApoE4 neurons and astrocytes are susceptible to SARS-CoV-2 infection (Wang et al., 2021). Moreover, post-mortem brain tissue analysis of a COVID-19 patient revealed the presence of viral particles in brain endothelial cells, indicating possible direct neurotropism (Bullen et al., 2020; Paniz-Mondolfi et al., 2020). Alternatively, SARS-CoV-2 neurotropism can exert its actions non-cell autonomously through the use of soluble mediators; extracellular vesicles; inflammatory and immune cell signaling; non-traditional neurotransmitter, neuropeptide and ion channel signaling; via the lymphatics; and by microvascular damage (Hanoun et al., 2015; Solomos and Rall, 2016; Benveniste et al., 2017; Maryanovich et al., 2018; Merad and Martin, 2020; Rhea et al., 2021). Because a relatively low rate of SARS-CoV-2 RNA is detected in the brain tissue of patients who died from COVID-19, there is a developing consensus that neurological manifestations associated with COVID-19 are due, in large part, to non-cell autonomous effects (Serrano et al., 2021). For example, of twenty COVID-19 patients studied in one neuro-anatomical survey, only two had unequivocal neuropathological findings, and only one of those two had detectable SARS-CoV-2 RNA in the brain (Serrano et al., 2021).

Neurotropism can be the result of blood-borne viruses subverting the blood-brain barrier (BBB) (Iadecola et al., 2020). SARS-CoV-2 has been shown to cross the BBB in a murine model (Rhea et al., 2021). Using the S1 subunit of the SARS-CoV-2 spike protein as a proxy for uptake, viral entry was observed throughout the mouse brain parenchyma and in endothelial cells (Rhea et al., 2021). These results implicate direct viral invasion of the BBB in SARS-CoV-2 mediated neurological dysregulation. ACE2 and other viral proteases are expressed on endothelial cells of the vasculature (Xiao et al., 2020). Because lung tissue infected with SARS-CoV-2 shows patterns of damage characteristic of pulmonary fibrosis, viral blood-borne tropism may be facilitated in COVID-19 through upregulated ACE2 in these arterial vascular cells (Guo et al., 2020). Immune cells reaching the CNS may also promote neurotropism, particularly via macrophages and monocytes (Merad and Martin, 2020). Although several autopsy studies have not revealed widespread immune cell infiltration, the potential contributions of infected innate and acquired immune cells to neurotropism may be consequential (Iadecola et al., 2020).

Once in the circulation, the virus may easily avoid the BBB by targeting circumventricular organs in the brain. These CNS sites have areas of fenestrated capillaries where the endothelial border does not represent a fixed boundary to molecular entry (Iadecola et al., 2020). The presence of ACE2 receptors in such delimited CNS locations support this potential route of entry (Chigr et al., 2020). Furthermore, inflammation resulting from reactive oxygen species and free radicals produced by SARS-CoV-2 infection can damage the BBB, facilitating neurotropism via the circulation in other locations lacking fenestrations (Li et al., 2020c). Similarly, cytokines, which are elevated during COVID-19 infection, are known to cross the BBB and contribute to neuroinflammation observed in SARS-CoV-2 brain neuropathological studies (Azizi and Azizi, 2020; Iba et al., 2020; Rhea et al., 2021).

Although the BBB largely segregates the brain from the systemic circulation, toxic wastes and metabolites must still be removed from the brain. A waste removal system, termed “glymphatics” for its dependence on glial cells, is facilitated by a CSF and interstitial fluid transport system (Benveniste et al., 2017). In this system, CSF is transported within a peri-vascular network that helps drain waste from the brain parenchyma (Benveniste et al., 2017). In addition to glymphatics, a true lymphatic vasculature that drains interstitial fluid has been described in the brain’s meningeal layer (Solomos and Rall, 2016). Furthermore, the brain dural sinuses are another location of potential neuro-immune interactions. At the dural sinus, accumulated CNS-derived antigens in the CSF are taken up by dural antigen-presenting cells that introduce the antigens to patrolling T cells (Rustenhoven et al., 2021). Circulating lymphocytes and viral proteins in these systems might be potential routes of both neuroinvasion and subsequent transport back to the periphery (Solomos and Rall, 2016). Other coronaviruses have previously been documented to be present in the CSF (Nath, 2020), and now SARS-CoV-2 has been observed in the CSF of COVID-19 patients with rare neurological presentations like acute necrotizing encephalopathy and demyelination (Domingues et al., 2020; Virhammar et al., 2020).

## Molecular and Cellular Mechanisms of Viral Entry and Signal Transduction

### Angiotensin Converting Enzyme 2/1 (ACE2/1)

The ACE2 receptor plays a pivotal role as the initiator of SARS-CoV-2 cellular entry in multiple organs (Xiao et al., 2020). When the viral spike glycoprotein interacts with ACE2, SARS-CoV-2 is endocytosed (Liu et al., 2020a). ACE2 is a key protein for the renin-angiotensin system (RAS), which plays a central role in the homeostatic regulation of electrolyte and fluid balance, blood pressure, arterial oxygenation, and end organ function, particularly those mediated by the renal and cardiovascular systems (Li et al., 2017; Miller and Arnold, 2019). The RAS is divided into vasopressor and vasoprotective axes (Li et al., 2017). The multiple arms of this complex pathway ultimately result in the dynamic interplay of two main enzymes: Angiotensin Converting Enzyme 1 (ACE1) and ACE2. ACE1 converts angiotensin I (Ang I) to angiotensin II (Ang II), while ACE2 converts Ang I and Ang II to angiotensin (1-7) (Ang (1-7)) and angiotensin (1-9) (Ang (1-9), respectively (Miller and Arnold, 2019). The vasopressor axes are comprised of the classic angiotensinogen/renin/ACE1/Ang II axis and the prorenin/renin axis (Li et al., 2017). The vasoprotective axes, which are dependent on ACE2/Ang (1-7)/Ang (1-9)/Mas receptors, counteract detrimental effects of the vasopressor axes (Li et al., 2017). When SARS-CoV-2 is endocytosed, the ACE2 enzyme is endocytosed with it, shifting the balance of the RAS toward the pro-inflammatory vasopressor axes (Li et al., 2017; Gheblawi et al., 2020). Such disruption of RAS balance, which has previously been implicated in the development of cardiovascular disease, renal disease, and hypertension, amongst other systemic derangements, may be a driver of COVID-19 systemic dyshomeostasis (Li et al., 2017).

### Protease Cofactors and Alternative Pathways

Proteases help to orchestrate neurotropism by assisting the ACE2 receptor interaction with SARS-CoV-2. For epithelial cells, these proteases may even assist viral invasion through non-ACE2-mediated routes, via CD147-spike protein and CD26 expressed ubiquitously (Radzikowska et al., 2020; Wang et al., 2020a). ACE2 receptors are further modulated by being endocytosed following binding to SARS-COV-2 (Gheblawi et al., 2020; Hirano and Murakami, 2020). Transmembrane protease serine 2 (TMPRSS2) is a serine protease on the plasma membrane that mediates spike protein activation and promotes SARS-CoV-2 entry via direct fusion, thereby subverting endocytic entry (Bailey and Diamond, 2021). ACE2 can exist as a transmembrane bound protein in vascular endothelial cells, or as a circulating form, once cleaved by TMPRSS2 or transmembrane protease serine 4 (TMPRSS4) (Xiao et al., 2020). ACE2 is subsequently shed, giving rise to the circulating form, while TMPRSS2 and TMPRSS4 simultaneously facilitate the endocytosis of SARS-CoV-2 (Xiao et al., 2020).

The SARS-CoV-2 spike protein has been shown to interact with the soluble form of ACE2 or a soluble ACE2-vasopressin complex extracellularly, and then may enter cells via endocytosis mediated by the angiotensin II type I receptor (AT_1_R) or arginine-vasopressin receptor 1B (AVPR1B), respectively (Yeung et al., 2021). Additionally, the soluble form of ACE2 preserves the viral binding site and may therefore facilitate viral tropism of cells where the tissue-bound form of ACE2 is poorly expressed (Yeung et al., 2021).

Furin is a pro-protein convertase found in many tissues, including the brain, neuroendocrine organs, the GI tract and liver, with few differences in expression levels (Wu et al., 2020). The SARS-CoV-2 spike protein contains a unique furin cleavage site not found in other β-coronaviruses (Wu et al., 2020). Furin cleavage facilitates stronger viral receptor binding and membrane fusion. These may contribute to the high infectivity profiles and the multi-organ involvement, especially where ACE2 is present at lower levels of expression (Wu et al., 2020). For example, furin produced in intestinal cells could provide an avenue for viral entry into the CNS via the myenteric nerve plexus. Moreover, loss of furin is an effective measure to prevent viral infection (Johnson et al., 2021). Other proteases like cathepsin L, cathepsin B, trypsin, factor X, elastase, and Coronavirus 3CL protease have also been implicated in SARS-CoV2 binding (Gheblawi et al., 2020; Macchiagodena et al., 2020). CRISPR-Cas-9 screening has also identified several genes necessary for the synthesis of glycosaminoglycans, which are negatively charged polymers that likely increase infectivity by attracting exposed positive charges on viruses (Bailey and Diamond, 2021).

Interferon modulation of ACE2 receptors can lead to increased degrees of neurotropism (Merad and Martin, 2020; Ziegler et al., 2020). Interferons are downstream inflammatory products of IL-1, IL-6, and tumor necrosis factor (TNF), key inflammatory molecules released by immune cells in multiple tissues affected by COVID-19 (Ziegler et al., 2020). Severe COVID-19 infection and death following infection have been associated with elevated inflammatory markers and chemokines (Merad and Martin, 2020). The resulting interferons enhance viral invasion as Ziegler et al. demonstrated that ACE2 receptors represent the translation product of interferon-stimulated genes in human barrier tissue epithelial cells (Ziegler et al., 2020). Smoking and chronic obstructive pulmonary disease (COPD) are associated with the increased presence of endocytic vacuoles implicated in SARS-CoV-2 endocytosis (Eapen et al., 2021). Patients with this pathogenic profile may be more susceptible to viral entry and associated profiles of neurotropism. With endocytosis and downregulation of surface ACE2 proteins, the pro-inflammatory axes of the RAS can prevail, namely through Ang II and the AT_1_R (Gheblawi et al., 2020). Interferon-stimulated ACE2 and ACE2 endocytosis both can modulate viral entry. However, a novel truncated isoform of the ACE2, termed deltaACE2 or MIRb-ACE2, has recently been discovered (Ng et al., 2020; Onabajo et al., 2020). This truncated ACE2 isoform has been observed to be induced by interferons and viruses, including SARS-CoV-2 (Ng et al., 2020; Onabajo et al., 2020). The novel truncated ACE2 isoform does not appear to bind the SARS-CoV-2 spike protein or act as a peptidase, suggesting that interferon-stimulated induction may not play a role in promoting SARS-CoV-2 cellular entry (Ng et al., 2020; Onabajo et al., 2020).

## Nervous System Contributions to Systemic Dyshomeostasis

Several key molecular factors are closely involved in stress-mediated dysregulation, including catecholamines: adrenaline, noradrenaline, dopamine (DA); peptide hormones and associated factors: arginine vasopressin (AVP), Ang II; and steroid hormones, including cortisol (Goldstein, 2020). This broad-based stress-mediated bioactive factor deregulation leads to activation of the sympathetic nervous system which, in turn, increases Ang II, depletes ACE2 and subsequently decreases the protective action of Ang (1-7) (Xiao et al., 2011; Porzionato et al., 2020). These mechanisms are associated with typically observed laboratory values and manifestations observed in COVID-19 infection: hyperglycemia, hyponatremia, electrocardiogram abnormalities, cytokine storm, heart failure, acute kidney injury, acute respiratory distress syndrome (ARDS), clotting disorders, and emotional stress (Porzionato et al., 2020).

### Distributed Renin-Angiotensin System

The Ang II peptide of the RAS can act on a few receptor types, the most notable being AT_1_R to effect change, including increased sympathetic tone (Xia and Lazartigues, 2010; Miller and Arnold, 2019). AT_1_R is a major mediator of RAS in raising blood pressure through several homeostatic mechanisms (Miller and Arnold, 2019). AT_1_R is localized at circumventricular organ sites and other cardinal integrative regulatory centers of the brain like the hypothalamus and medulla (Doobay et al., 2007). Circulating Ang II can mediate activation of the SNS by acting on circumventricular organs and the carotid body (Porzionato et al., 2020). This system is triggered during times of blood loss, hyponatremia, renal hypotension, general SNS activation and infection. Notably, an overactive SNS is implicated in many comorbidities associated with mortality in COVID-19 (Porzionato et al., 2020). Ang II interacts with the pro-inflammatory AT_1_R, and downstream products of these processes activate STAT3 and NF-κB transcription factors (Hirano and Murakami, 2020). This promotes IL-6 production, which upregulates NF-κB and STAT3 with production of other pro-inflammatory cytokines (Hirano and Murakami, 2020).

ACE2 allows Ang (1-7) to act on Mas receptors to countervail AT_1_R actions (Miller and Arnold, 2019), since the Mas receptor mediates vasodilation and anti-proliferative effects (Xia and Lazartigues, 2010). ACE2 overexpression promotes a protective phenotype in cardiovascular disease states in transgenic mouse models (Alenina and Bader, 2019). ACE2 in the hypothalamus has powerful antihypertensive and sympathetic nervous system dampening effects, with ACE2 overexpression increasing Ang (1–7) relative to Ang II, thereby providing beneficial effects in mouse models displaying brain injury and stroke (Alenina and Bader, 2019). ACE1/ACE 2 receptor interactions work to effectively mediate the effects of Ang II and Ang (1-7). Unfortunately, with SARS-COV-2 infection, AT_1_R activity frequently prevails.

### ACE2-Mediated Cellular Tropism and Organ System Damage

As mentioned, the SARS-CoV-2 spike protein interacts with the ACE2 receptor in epithelial cells, which line a variety of tissues including the lung, heart, and kidney. SARS-CoV-2 infection thus potentially contributes to intricate profiles of end organ damage via interacting neural and ACE2-RAS deregulation of cardinal homeostatic processes. The involvement of and damage to the CNS affects different systemic organ systems which then exert feedback regulation to the CNS to exacerbate the neurological and overall clinical patterns of COVID-19 infection (Robinson et al., 2016; Batlle et al., 2020; Garvin et al., 2020; Hundt et al., 2020; Lei et al., 2020). Recent autopsy results of COVID-19 patients reflect dysregulation of key molecular factors involved in hypoxia, coagulation, and fibrosis in multiple organs, highlighting the extent of aberrant local regulatory processes and systemic homeostasis (Nie et al., 2021).

#### Lung Injury

Pulmonary edema as a result of SARS-CoV-2 infection may occur as a result of the activation of the bradykinin 1 and 2 receptors (B1R and B2R, respectively) in lung epithelial cells. Normally, ACE2 plays a protective role by preventing fluid accumulation through inactivation of the B1R ligand known as bradykinin (BK). Bradykinin lowers blood pressure and promotes capillary leakage. Furthermore, elevated D-dimer levels in these patients are most likely reflective of the leakage of plasma contents. A similar mechanism leading to pulmonary edema has been suggested to play an important role in ARDS and COVID-19 due to increased hydrostatic pressures as a result of increased Ang II. Increased Ang II has been experimentally shown to not alter hydrostatic pressure; however, increased bradykinin has been proposed to increase hydrostatic pressure. ACE1 inactivates bradykinin, which is an activating ligand of B2R, while ACE2 inactivates nine-arginine bradykinin (Arg9-BK) and bradykinin without nine-arginine (des-Arg9-BK) which are activating ligands of B1R (van de Veerdonk et al., 2020). In bronchoalveolar samples of COVID-19 patients, the BK precursor, kininogen, was expressed while remaining undetected in control specimens. Furthermore, BK degradative enzymes were downregulated compared to those present in control patients (Garvin et al., 2020). There is significant upregulation of the genes encoding the enzymes responsible for production of different forms of hyaluronic acid. The corresponding enzymes responsible for its degradation, hyaluronidases, are conversely downregulated. Hyaluronic acid promotes avid water absorption leading to the formation of a hydrogel within the lungs. The combination of increased vascular permeability due to increased bradykinin levels as well as increased hyaluronic acid production leads to hydrogel formation impairing the diffusion of oxygen and carbon dioxide within the lungs of patients with COVID-19. Interestingly, the alveolar type I cells in the lung may act as homeostatic regulators of alveolar organization and function following injury, since they have been described to remodel the postnatal mice alveolus via distinct intracellular signaling factors (Zepp et al., 2021).

#### Liver Injury

Although the exact mechanisms of liver damage in COVID-19 remain to be elucidated, direct tropism via the ACE2 receptor expressed in hepatocytes, secondary hepatic damage as a result of systemic inflammation, and the hepatotoxicity of COVID-19 therapies contribute to organ dysfunction (Han et al., 2021a). Because the liver plays an important role in regulating inflammation by producing acute phase proteins, complement proteins, and several cytokines, liver damage in COVID-19 could potentially contribute to central homeostatic dysregulation of immune function (Robinson et al., 2016).

#### Kidney Injury

The kidney regulates blood pressure and blood volume through the RAS. Acute kidney injury (AKI) is frequently observed in hospitalized COVID-19 patients (Batlle et al., 2020; Benedetti et al., 2020; Farouk et al., 2020; Sharma et al., 2020). As with COVID-19-associated liver injury, the mechanisms of AKI are likely multifactorial. ACE2 is strongly expressed in the kidney, suggesting direct SARS-CoV-2 renal tropism (Batlle et al., 2020; Benedetti et al., 2020; Farouk et al., 2020). However, the presence of SARS-CoV-2 RNA and viral particles in the kidney has not been consistently reported in the literature (Farouk et al., 2020; Sharma et al., 2020). The non-cell autonomous influences of hyperinflammation, hypoxemia, and hypercoagulability may contribute to the development of AKI (Batlle et al., 2020; Benedetti et al., 2020; Farouk et al., 2020). Moreover, ACE2 expression has been found to be downregulated in AKI (Batlle et al., 2020). This may lead to higher ACE1 expression and lower levels of Ang (1-7), which could promote a hyperinflammatory and hypercoagulable state in the kidney (Batlle et al., 2020). Because the causes of COVID-19 AKI are likely multifaceted, the disruption of RAS homeostasis in the kidney may create a vicious cycle that exacerbates renal and systemic damage.

### Cardiorespiratory Failure and Hypoxemia

SARS-CoV-2 has been hypothesized to infect the CNS through the cribriform plate, travel in a retrograde fashion through peripheral olfactory nerves to the brain stem (inclusive of the medulla) and to target and destroy the medullary centers responsible for cardiorespiratory control, such as the Pre-Botzinger complex. This has been thought to result in the compromise of the respiratory centers (Gandhi et al., 2020). Similar neurotropic viruses, like the avian coronavirus, have been reported to track to nuclei of the medulla: the NTS and the nucleus ambiguus (NA), sites that receive information from the chemoreceptors of the respiratory tract and lungs and innervate local resident smooth muscle, glands, and vessels (Costa et al., 2014; Li et al., 2020b).

In SARS-CoV-2 infection, there is a proposed loss of homeostatic control by the medulla resulting in cardiorespiratory compromise: inadequate blood flow, respiration and oxygenation, leading to a multifaceted impairment of alveolar gas exchange. In healthy individuals, peripheral chemoreceptors, present at highest concentrations in the carotid body and aortic arch, monitor the flux of arterial oxygen levels. When there is arterial oxygen desaturation, an excitatory signal is sent from the glomeruli here, most directly to the NTS, integrating at the rostral ventrolateral medulla (Costa et al., 2014). In a hypoxic state with low oxygen reserves, the body will aggressively work to increase blood flow to high impact organs, promote vasoconstriction in other peripheral locations, and increase respiratory rate as well as inspiratory/expiratory force, in an attempt to efficiently deploy oxygen (Costa et al., 2014; Tassorelli et al., 2020). There is likely an inability to adequately detect and respond to changes in oxygenation when SARS-CoV-2 infection compromises medullary and carotid body oxygen sensing and set-point regulation as well as sensory nerve-related respiratory muscle function. The often profound hypoxemia, frequently without premonitory symptoms, is characteristic of COVID-19 patients facing imminent respiratory compromise and is indicative of homeostatic deregulation.

Interestingly, increases in peripheral hypoxic chemosensitivity have been ascribed to overstimulation of the sympathetic nervous system (Porzionato et al., 2020). A hyperactive sympathetic nervous system due to higher hypoxic chemosensitivity has been implicated in chronic obstructive pulmonary disorder and metabolic syndrome, comorbidities that enhance the severity of COVID-19 (Porzionato et al., 2020).

### Gastrointestinal Dysfunction

Acute GI symptoms of COVID-19 include nausea, vomiting, and diarrhea (Esposito et al., 2020; Li et al., 2020c). Viral neurotropism of the area postrema and in the NTS of the medulla oblongata of the brainstem may disrupt their normal homeostatic roles. The NTS is particularly known to be targeted by other coronaviruses, emphasizing its potential role in the acute GI symptoms frequently seen in COVID-19 (Porzionato et al., 2020).

The ENS regulates gut homeostasis through interactions between enteric glial cells, gut epithelium, and gut-associated lymphoid tissue (GALT) (Esposito et al., 2020). Enteric glial cells serve as antigen-presenting cells to the GALT, and their activation is characterized by IL-6 release, contributing to inflammation in COVID-19 (Esposito et al., 2020). Furthermore, their activation by viruses has been implicated as a key step in neurological immune priming that leads to later neurological impairments (Esposito et al., 2020). Deregulation of these components of the nervous system may therefore be responsible for the observed GI dysfunction.

### COVID-19 Associated Coagulopathy

Specific forms of neurological dysfunction in COVID-19 may be related, among other pathological processes, to characteristic features of the newly-defined COVID-19 associated coagulopathy (CAC). Disruption of interactions between the coagulation cascade and inflammation has been observed in SARS-CoV-2 infected patients; CAC is unique in that it presents with elevated fibrinogen, D-dimer levels, Von Willebrand factor (VWF), factor VIII and inflammatory cytokines that can induce a generalized thrombotic disorder (Iba et al., 2020). SARS-CoV-2 infected macrophages express tissue factor on their cell surface promoting coagulation. Factor VIII and VWF released by SARS-CoV-2 infected endothelial cells also contribute to increased coagulation. Thrombosis may occur due to these multifaceted prothrombotic modifications in cell signaling (Iba et al., 2020). However, unlike other coagulopathies, CAC is not marked by thrombocytopenia and does not show increased partial thromboplastin time, distinguishing it from similar disorders of sepsis-induced coagulopathy (Iba et al., 2020).

CAC is notable for endothelial damage as SARS-CoV-2 directly infects vascular endothelial cells via ACE2 receptors with the help of TMPRSS2 cleavage. Damaged endothelial cells then fail to produce nitric oxide, thus allowing adhesion of leukocytes and platelets and migration of inflammatory cells into the vessel wall (Iba et al., 2020). In the event of infection, endothelial cell damage results in tissue factor release, especially in the brain, activating thrombin, the final serine protease in the coagulation cascade (Festoff and Citron, 2019). Because thrombin exerts pro-inflammatory effects in addition to its role in coagulation, it plays a key role in the interactions mediating the coagulation-inflammatory nexus (Festoff and Citron, 2019). Thrombin cleaves the protease-activated receptor on endothelial cells to gain access through the BBB to the CNS, where it can then lead to neuroinflammation (Festoff and Citron, 2019).

The result of this robust coagulation, inflammation, and endothelial damage, including active viral-mediated endotheliitis, is profound degrees of vasculitis, thrombosis, and stroke affecting large arterial and venous vessels as well as the entire brain and body microvasculature, including arterioles, venules and capillary beds. This results in complex impairments in oxygen exchange and unique microinfarctions and mechanosensitive microbleeds with dramatic CNS microglial activation, active innate immune sensing and neuroinflammation (Azizi and Azizi, 2020; Iba et al., 2020; Thakur et al., 2021). Microscopic examination of the brains of patients who have died from COVID-19 has indicated multifocal microvascular ischemic and hemorrhagic parenchymal injury, neuroinflammation and microglial activation without cell autonomous viral effects targeting neurons, astrocytes or oligodendrocytes (Lee et al., 2020; Thakur et al., 2021).

### Neural-Immune, System Inflammatory and Hematopoietic-Mediated Dysfunction

An unusual inflammatory syndrome characterized by fever, inflammation, and multisystem organ injury has been associated with a subset of COVID-19 patients, including children (Whittaker et al., 2020). Elevated fractions of mononuclear phagocytes, particularly inflammatory monocyte-derived macrophages, have also been observed in COVID-19 patients (Liao et al., 2020; Merad and Martin, 2020). Overactivation of these mononuclear phagocytes may contribute to the cytokine storm that is characteristic of COVID-19, and the cytokine profile of COVID-19 hyperinflammation resembles that of macrophage activation syndrome (Merad and Martin, 2020). Elevated cytokines, namely IL-6 and tumor necrosis factor (TNF), released by macrophages in severe SARS-CoV-2 infection, not only promote an inflammatory milieu, but may also stimulate upregulation of the ACE2 receptor, further driving SARS-CoV-2 regional cellular tropism (Merad and Martin, 2020).

Hyperinflammation and activation of mononuclear phagocytes may also contribute to the process of hypercoagulation observed in COVID-19. In the absence of direct vascular damage, the initiation of coagulation is dependent on increased expression of the pro-thrombotic molecule, coagulation factor III, on mononuclear cells via pro-inflammatory cytokines (Merad and Martin, 2020). An inflammatory state inhibits anticoagulant pathways, further promoting a severe systemic hypercoagulable state. For example, antiphospholipid antibodies, which are characteristically present in several autoimmune conditions, activate the complement and coagulation cascades to contribute to thrombosis and to mediate proinflammatory signaling in innate immune and vascular endothelial cells (Muller-Calleja et al., 2021). Müller-Calleja et al. recently described a process by which endothelial protein C receptors act in complex with the lipid lysobisphosphatidic acid to activate pathogenic antiphospholipid antibodies, representing a mechanistic link between coagulation and innate immune signaling (Muller-Calleja et al., 2021).

The autonomic nervous system plays a crucial homeostatic role through its pro-inflammatory and anti-inflammatory effects on immune cells. An innate immune response activates sensory neurons that release neuropeptides such as calcitonin-gene-related peptide and substance P, the downstream products of which promote inflammation (Hanoun et al., 2015). When the HPA axis detects inflammation via afferent nerves or inflammatory markers, it exerts an anti-inflammatory effect by stimulating glucocorticoids and dopamine release from the adrenal glands (Hanoun et al., 2015; Maryanovich et al., 2018).

Both major branches of the autonomic nervous system contribute to neural regulation of the immune response. The SNS releases noradrenaline that exerts distinct actions through specific subtypes of adrenergic receptors. Noradrenaline has pro-inflammatory effects at low concentrations and anti-inflammatory effects at high concentrations (Hanoun et al., 2015). Chronic inflammation alters the expression pattern of adrenergic receptor subtypes and shifts local innervation toward pro-inflammatory sensory nerves (Hanoun et al., 2015); it is possible that COVID-19 causes this type of shift as well (Porzionato et al., 2020). Furthermore, in murine models, increased sympathetic stimulation led to noradrenaline-mediated vascular inflammation and increased output of neutrophils and inflammatory monocytes (Porzionato et al., 2020). While there is no definitive evidence for direct parasympathetic innervation of the immune system, inflammatory cytokines activate afferent branches of the parasympathetic NS to send signals to the NTS and activate efferent branches of the parasympathetic NS (Hanoun et al., 2015; Maryanovich et al., 2018). The parasympathetic NS can then counteract an inflammatory state via acetylcholine release (Hanoun et al., 2015). For example, activation of the parasympathetic NS has been shown to mediate an anti-inflammatory immune cell response via inhibition of tumor necrosis factor release by macrophages (Porzionato et al., 2020).

The circadian control of the immune system represents an additional axis that may be disrupted by COVID-19. The “master clock” of the circadian system is the suprachiasmatic nucleus of the hypothalamus (Scheiermann et al., 2013). The HPA axis and SNS modulate local circadian rhythms in body tissues through hormonal and autonomic neural regulation of peripheral clock components (Scheiermann et al., 2013). The SNS can act as a local regulator of the body clocks because the SNS directly innervates tissues and drives cyclical noradrenaline release from nerve varicosities (Scheiermann et al., 2013). A large complement of circulating hematopoietic cells, lymphocytes, hormones, and cytokines have been shown to exhibit circadian oscillations (Scheiermann et al., 2013). In murine models, the acute inflammatory response exhibits circadian rhythms, likely due to circadian-influenced leukocyte migration and phagocytic activity (Scheiermann et al., 2013). Furthermore, several diseases in humans display circadian dysregulation as part of their presentation. Myocardial infarction and ischemic stroke, documented consequences of COVID-19, are examples of such diseases, where a surge in SNS activity during the morning hours is thought to contribute to cardiovascular complications (Scheiermann et al., 2013; Choudry et al., 2020). It is conceivable that SARS-CoV-2 neurotropism, both in the central and peripheral nervous systems, could therefore be adversely impacting the modulation of the circadian system over the immune system, thereby contributing to some of the observed COVID-19-related acute phase complications.

Furthermore, the peripheral nervous system also dynamically regulates bone remodeling and bone marrow effector functions. Hematopoietic stem cells (HSCs) in the bone marrow are activated in response to infection to maintain homeostasis (Maryanovich et al., 2018). The SNS has been documented to regulate the microenvironment in the bone marrow, where sympathetic nerve fibers help to create niches essential for normal HSC egress and differentiation of individual blood elements as well as behavioral effects during stress (Maryanovich et al., 2018). Sensory nerves also reach the bone marrow, and sensory neuropeptides have been suggested to directly contribute to regulating HSC activity (Maryanovich et al., 2018). Many of the unique systemic manifestations of COVID-19 affect the derivatives of HSCs like platelets, red blood cells, and immune cells, potentially suggesting that HSCs represent another end organ effector system at the cellular level whose components are differentially regulated and impaired by SARS-CoV-2.

Bidirectional neuro-immune communications involving the lymph nodes represent another axis where the function of sensory neurons may be disrupted. Recent work by Huang et al. further elucidates previously described communications between the PNS and the immune system (Huang et al., 2021). They find that lymph nodes are innervated by heterogeneous populations of sensory and sympathetic neurons which demonstrate inflammation-induced plasticity (Huang et al., 2021). These sensory neurons are largely skewed toward peripheral lymph nodes, suggesting that these neurons can act to monitor the homeostatic status of the lymphatic system (Huang et al., 2021). When activated, these neurons can modulate activity-dependent gene transcription in immune cells (Huang et al., 2021). SARS-CoV-2 neurotropism could be differentially targeting sensory neurons in this heterogeneous population, thereby contributing to the maladaptive immune response. Dysregulation of the interactions of the PNS with the immune system may therefore be contributing to the hyperinflammatory state of COVID-19 and its concomitant and protean systemic manifestations.

## Long-Term Neurological Sequelae

There is increasing evidence of a “Long COVID” syndrome, in which some COVID-19 patients, regardless of initial disease severity, experience manifestations of the illness beyond acute COVID-19 infection (Al-Aly et al., 2021; Boldrini et al., 2021). A profile of patients with “Long COVID” reveals fatigue, post-exertional malaise, and cognitive dysfunction as the most common symptoms of this syndrome reported up to 6 months from the acute period of illness (Davis et al., 2020). Many of these patients experience prolonged multisystem involvement and significant disability (Davis et al., 2020). This is analogous to a prolonged post-concussive syndrome associated with elements of traumatic brain injury. Such a clinical course is compatible with the lack of regenerative responses described in COVID-19 (Delorey et al., 2021).

Given the unprecedented range and severity of acute neurological COVID-19 syndromes already described in the literature, the consequences of prolonged aberrant, viral-mediated neural regulation of systemic processes are likely to be profound. Many of the observed long-term sequelae are likely due to a disruption of the bi-directional interactions of the nervous system and the immune system detailed above, which promotes a pro-inflammatory, hypoxemic, hypercoagulable state. This sustained detrimental environment is known to promote long-term neurological syndromes including demyelinating disorders, degenerative dementias, movement disorders, delayed immune-mediated encephalopathies, neuromuscular disorders, neuropsychiatric conditions and unusual cognitive disorders frequently seen with prior delayed effects of head and spinal cord injuries, rare focal neurovascular syndromes, and more remote effects of viral encephalitides (Tavassoly et al., 2020). These post-acute sequelae may be associated with alterations in cortical circuit hyperconnectivity, impaired excitation-inhibition coupling, excitotoxicity, and immune memory dysfunction (Brun et al., 2020; Domingues et al., 2020; Zanin et al., 2020).

The resulting pathological substrate, including COVID-19 coagulopathy, vasculopathy, neuroinflammation, and immune dysregulation, increases the risk of COVID-19 related strokes, including atypical presentations indicative of multiple large vessel involvement or diffuse microvascular ischemia and hemorrhage, the latter frequently presenting as a persistent encephalopathy without additional lateralizing neurological signs representing the potential progression of a classical delirium into a syndrome of mild to moderate protracted cognitive disability (Hess et al., 2020). Additionally, demyelinating lesions of the CNS have been described as a sequela as a result of inflammation-induced glial cell activation (Brun et al., 2020; Zanin et al., 2020). Similarly, neuronophagia and microglial nodules have been found in the cerebellar dentate nuclei of a COVID-19 patient (Al-Dalahmah et al., 2020). These may be the result of microglial cell activation by cytokines, which drives phagocytosis of hypoxic neurons (Al-Dalahmah et al., 2020). ApoE4, a genetic risk factor for neural injury responses in Alzheimer’s disease and other degenerative dementias and chronic traumatic encephalopathy, has been shown in glial cells to exacerbate neurodegeneration and is associated with more severe COVID-19 (Wang et al., 2021). SARS-CoV-2 has been shown to preferentially target and induce death of human induced pluripotent stem cell-derived ApoE4 astrocytes (Wang et al., 2021). These results may help explain why a subset of COVID-19 patients have neurodegenerative manifestations and accelerated aging phenotypes (Wang et al., 2021).

## Systemic Viral Sequelae

The inability to return to normal homeostasis after acute infection may result in an unprecedented level of stress and injury, which has profound implications for the long-term sequelae of COVID-19. In this section, we explore how this unique pathology may lead to organ fibrosis, tissue degeneration, and systemic cancer.

Multiple cellular responses must be coordinated to restore homeostasis (You et al., 2021). Severe stress conditions adversely impact the differentiation and tissue regenerative functions of HSCs (Cho et al., 2013). In human stress and disease environments, transcriptional and post-transcriptional mechanisms disrupt tissue-specific stem and progenitor cell homeostasis (Puisieux et al., 2018). For example, dysregulation of the mitochondrial unfolded protein stress response impairs HSC proliferation, and additional degrees of extreme stress via the endoplasmic reticulum (ER) stress response induces HSC apoptosis and associated regenerative responses (Puisieux et al., 2018). Moreover, sympathetic hyperactivation, a feature of COVID-19, has been observed to deregulate HSC niches (Maryanovich et al., 2018).

Stress and injury also have influences at the level of inter-organelle communications, where, for example, stress to the ER has been identified as a hallmark of hyperinflammation, protein misfolding, and cell death (You et al., 2021). The unfolded protein response is central to the resolution of ER stress programs because it modulates newly-described transcriptional regulators like QRICH1 that dictates cell fate responses (You et al., 2021). In situations of unresolved, sustained stress, a dysregulated unfolded protein response can ultimately induce cell dysfunction and death (You et al., 2021). In *C. elegans* models, dopaminergic and serotonergic neurons have been found to be necessary and sufficient for activating the non-cell autonomous ER-mediated unfolded protein response in peripheral tissues to promote lipid homeostasis, including lipophagy, and proteostasis that extend lifespan (Daniele et al., 2020; Higuchi-Sanabria et al., 2020). These results emphasize the dynamic interplay between the nervous system and peripheral homeostatic mechanisms in responding to stress to regulate the continuum between premature aging phenotypes and longevity responses. Nuclear histones are another cellular component whose role in suppressing immunogenicity has been highlighted (Uggenti et al., 2020). Stress and injury may interfere with the ability of specific components of the immune system to distinguish between self- and non-self-nucleic acids, as emphasized by autoinflammatory disorders associated with deregulated type I interferon responses (Uggenti et al., 2020). Chromatin without linker histones stimulates the DNA sensor, cGAMP, activating the interferon response which ultimately leads to self-DNA-induced immunogenicity and inflammation (Uggenti et al., 2020).

Epithelial cells in multiple organs participate in tissue regenerative capabilities that may be compromised in COVID-19 (Delorey et al., 2021). In the liver, both hepatocytes and biliary tract cells demonstrate regenerative plasticity, suggesting that these cells can act as facultative stem cells to replenish liver parenchymal cells following injury. A unique population of airway basal stem cells that can sense hypoxia has recently been identified (Shivaraju et al., 2021). Hypoxic conditions cause these stem cells to differentiate into solitary neuroendocrine cells, which can then mitigate the effects of hypoxic injury (Shivaraju et al., 2021). Solitary neuroendocrine cells secrete calcitonin gene-related peptide which acts as a paracrine signal to repair epithelial cell damage (Shivaraju et al., 2021). Damage to this protective cell type may lead to long-term airway epithelial cell damage. Epithelial cells in the lung in particular are important in recovery from pulmonary fibrosis (Basil et al., 2020). Alveolar epithelial type 2 (AT2) cells can act as a self-renewing stem cell-like population and regenerate alveolar epithelial type 1 cells, which constitute the majority of the alveolar surface (Basil et al., 2020). In pulmonary fibrosis, it has been hypothesized that stress and injury disrupt AT2 cell homeostasis and causes them to lose their regenerative capacity (Basil et al., 2020). If such a mechanism occurs with lung fibrosis observed in COVID-19, then the ability of the lungs to recover may be chronically compromised.

Potential damage to endothelial cells in the lymphatic system may play a role in the recovery from COVID-19 vasculopathy. Lymphatic endothelial cells have recently been found to secrete a lymphoangiocrine signal, the extracellular protein reelin (RELN) (Liu et al., 2020b). RELN mediates the proliferation of cardiomyocytes during mouse heart development and improves cardiac regeneration in neonatal mice (Liu et al., 2020b). In murine models, RELN has been shown to be essential for efficient heart repair and function following myocardial infarction (Liu et al., 2020b). RELN mediates the proliferation of cardiomyocytes. While it is unknown whether COVID-19 targets lymphatic endothelial cells, damage to this axis may present with disruption of putative tissue regeneration in the context of ongoing and possibly latent viral-mediated injury responses. Additionally, disruption of the glymphatic system has recently been suggested as a contributory factor to the progression of neurodegeneration that is likely operative in COVID-19 (Nedergaard and Goldman, 2020). The glymphatic system serves to clear protein aggregates during sleep; since age is associated with increased protein aggregation, decreased sleep quality, and risk for neurodegenerative disease (Nedergaard and Goldman, 2020); disruption of the glymphatic system may be a link between these deregulated homeostatic processes in COVID-19.

Organ fibrosis is a deregulated tissue regenerative response that can occur as a result of sustained or severe injury or chronic inflammation that is seen in COVID-19 (Henderson et al., 2020). During normal tissue repair following injury, local tissue fibroblasts are activated to synthesize components of the extracellular matrix and to secrete inflammatory mediators (Henderson et al., 2020). Deregulation of this cardinal homeostatic process, which may occur as a plausible consequence of COVID-19, can result in the excessive deposition and accumulation of a disorganized extracellular matrix within end organs (Henderson et al., 2020). These fibrotic changes fundamentally alter tissue structure, disrupt organ function, and can lead to organ and subsequent organismal dysfunction and death (Henderson et al., 2020). A persistent low level of stress due to COVID-19 may lead to a unique form of pan-organ fibrosis in which the organs do not actually undergo acute injury or death, but rather experience chronic dysfunction and novel forms of delayed brain and body malfunction (Henderson et al., 2020). There is increasing evidence that if the underlying cause of injury is removed, human organ fibrosis can resolve via the elimination of fibroblasts and the degradation of the extracellular matrix (Jun and Lau, 2018). In this way, the affected tissue does not progress to organ failure, but rather retains a degree of dysfunction and plasticity that would normally be observed in the initial or plateau phase of fibrosis. Moreover, recent analysis of single-cell atlases of multiple COVID-19 affected tissues has uncovered several failed tissue regenerative pathways in response to SARS-CoV-2, including the proliferation of fibroblasts and impaired AT2 differentiation (Delorey et al., 2021). This analysis also linked related cell types and genes implicated in disease severity to heritable risk using genome-wide association studies (GWAS) (Delorey et al., 2021), further underscoring how COVID-19 may act as an accelerated aging phenotype.

Stress and injury can also increase the susceptibility of cells to undergo malignant transformation (Puisieux et al., 2018). Environmental contexts such as pro-inflammatory signals have been suggested to affect cellular differentiation processes that protect against tumorigenesis (Puisieux et al., 2018). In inappropriate contexts like severe stress, differentiated adult epithelial cells undergo reprogramming, which may lead to the generation of cell states prone to malignancy (Puisieux et al., 2018). Central to this mechanism is the inactivation of p53, the loss of which promotes evasion of cell-cycle checkpoints and apoptosis (Puisieux et al., 2018). These processes also lead to enduring lineage infidelities and the commissioning of tumor related super-enhancers (Ge et al., 2017). Furthermore, Li et al. show that pancreatic inflammation is linked to a transient metaplastic progenitor cell population harboring a proto-oncogene mediated enhancer network that is co-opted by mutant Kras to subsequently drive tumorigenesis, suggesting that inflammation can also promote oncogenesis by gene enhancer network remodeling (Li et al., 2021). Moreover, innate immune priming and associated immune modulatory states can occur as a result of deregulation of specific immune molecular effector stress response pathways and result in a spectrum of context-specific outcomes including multiple forms of programmed cell death such as apoptosis, necroptosis, pyroptosis, inflammatory states, autoimmunity as well as immune deficiency with profound implications for intermediate and more long-term COVID-19 sequelae (Li et al., 2019; Lalaoui et al., 2020; Malireddi et al., 2020). Additionally, tumor cells can promote a hypercoagulable state by producing and secreting prothrombotic factors and inflammatory cytokines (Caine et al., 2002). Malignant transformation may therefore exacerbate CAC and lead to long-term coagulopathy. Taken together, the multiple modes and degrees of disruption in COVID-19 will likely subvert the body’s ability to maintain or reestablish homeostasis.

## Treatment Options

Repurposing drugs that can act on the autonomic and central nervous systems, including those targeting neurotransmitter and neuropeptide receptors and ligands that modulate immune cell subsets involved in COVID-19 immune dysregulation, may be an important direction for therapy. This would provide a route of symptom management and for halting disease progression through the application of already tested, manufactured, and inexpensive drugs. For example, hyperactivation of the SNS can be addressed using therapeutic agents for the adrenergic system. Further highlighting the opportunities for drug repurposing, imatinib, mycophenolic acid, and quinacrine dihydrochloride, which are already approved by the US Food and Drug Administration (FDA), have been shown to inhibit SARS-CoV-2 entry into relevant lung and intestinal cell types derived from human pluripotent stem cell-derived model systems (Han et al., 2021b). Moreover, recent analysis of the structural elements of the SARS-CoV-2 genome has identified specific host cellular proteins that bind to viral RNA and therefore may be targeted employing antisense oligonucleotides and existing FDA-approved drugs to reduce SARS-CoV-2 infection (Sun et al., 2021).

Addressing the long-term systemic sequelae will likely require sophisticated combination therapies. Depending on the temporal characteristics of a patient’s COVID-19 disease progression, immune agents that dampen or activate the immune system may be used, including advanced forms of checkpoint therapy for better optimization of cancer immunotherapy. Recently, *N*^6^-methyladenosine, an epigenetic modification of RNA involved in immune responses and tumorigenesis, has been found to activate the adenosine A3 receptor (Ogawa et al., 2021). The adenosine A3 receptor is a regulator of seminal tissue functions found to be overexpressed in inflammatory and cancer cells (Borea et al., 2015). This suggests that RNA modifications could be leveraged as a potential therapy for COVID-19 sequelae. Because COVID-19 potentially deregulates HSC niches, drugs affecting blood components may be potentially repurposed. For example, erythropoietin could be used to stimulate red blood cells and to mitigate hypoxemia (Soliz et al., 2020). The inflammatory environment created by neutrophils and neutrophil extracellular traps (NETs) have been implicated in tumorigenesis (Xiao et al., 2021). Xiao et al. have shown that targeting the tumor-secreted protease cathepsin C inhibits metastasis by limiting neutrophil recruitment and NET formation (Xiao et al., 2021), suggesting neutrophils are another blood component that can be regulated to mitigate the development of malignancy as a long-term sequela. Moreover, because remodeling of bone marrow niches can facilitate immune evasion and survival of malignant hematopoietic cells (Méndez-Ferrer et al., 2020), the microenvironment of these niches can also be targeted (Ho et al., 2019). Additionally, retrograde signals, including neurotransmitters, neuropeptides, hormones, and non-coding RNAs, by which the nervous system separately modulates lipid homeostasis, proteostasis and mitochondrial integrity in peripheral tissues through multiple mechanisms, including the integrated stress response and the mitochondrial unfolded protein response (Daniele et al., 2020; Higuchi-Sanabria et al., 2020), may therefore be repurposed to prevent premature aging phenotypes. This may also promote homeostatic responses underlying longevity to protect against a plethora of age-associated diseases likely to be seen as sequelae of COVID-19.

## Conclusion

There are many unaddressed questions that will impact the direction and manifestations of long-term COVID-19 sequelae. These include: How do the selective pressures driving SARS-CoV-2 mutational variants impact the interactions between the nervous system and systemic organs, tissues, and cell types? Does the spectrum of atypical systemic presentations and the prevalence of acute neuropsychiatric manifestations indicate the occurrence of rapid evolutionary forces influencing viral-mediated systemic and nervous system associated cross-regulation and dyshomeostasis? Are the long-term sequelae a consequence of ongoing effects of the acute disease state and/or distinct effects of injury responses, immunological memory, or latent reservoirs of SARS-CoV-2? Will the selective pressures that give rise to viral variants affect the long-term course of the COVID-19 disease process? Are SARS-CoV-2 variants inherently circumscribed by the evolutionary biology or rather are they infinitely adaptable?

## Author Contributions

HA and MFM conceptualized the proposed pathogenic mechanism, identified appropriate reference citations and participated in drafting and revising the manuscript. VE and GS identified appropriate reference citations and contributed to drafting the manuscript. IQ and TD provided important intellectual insights. All authors contributed to the article and approved the submitted version.

## Conflict of Interest

IQ was employed by the company Biohaven Pharmaceuticals. The remaining authors declare that the research was conducted in the absence of any commercial or financial relationships that could be construed as a potential conflict of interest.

## Publisher’s Note

All claims expressed in this article are solely those of the authors and do not necessarily represent those of their affiliated organizations, or those of the publisher, the editors and the reviewers. Any product that may be evaluated in this article, or claim that may be made by its manufacturer, is not guaranteed or endorsed by the publisher.
